# Tuneable local order in thermoelectric crystals

**DOI:** 10.1107/S2052252521005479

**Published:** 2021-06-30

**Authors:** Nikolaj Roth, Jonas Beyer, Karl F. F. Fischer, Kaiyang Xia, Tiejun Zhu, Bo B. Iversen

**Affiliations:** a Department of Chemistry and iNano, Aarhus University Aarhus, 8000, Denmark; b State Key Laboratory of Silicon Materials, School of Materials Science and Engineering, Zhejiang University, Hangzhou 310027, People’s Republic of China

**Keywords:** local order, diffuse scattering, thermoelectrics, hidden phases

## Abstract

Although crystalline solids are characterized by their periodic structures, some are only periodic on average and deviate on a local scale. Such disordered crystals with distinct local structures have unique properties arising from both collective and localized behaviour. Different local orderings can exist with identical average structures, making their differences hidden to Bragg diffraction methods. Using high-quality single-crystal X-ray diffuse scattering the local order in thermoelectric half-Heusler Nb_1−*x*_CoSb is investigated, for which different local orderings are observed. It is shown that the vacancy distribution follows a vacancy repulsion model and the crystal composition is found always to be close to *x* = 1/6 irrespective of nominal sample composition. However, the specific synthesis method controls the local order and thereby the thermoelectric properties thus providing a new frontier for tuning material properties.

## Abstract 2

Distinct ‘hidden’ phases of a technologically relevant thermoelectric material, which are identical in terms of composition and periodic crystal structure, but differ on a local scale, are observed and can be controlled through synthesis conditions. The local structure is explained in terms of a vacancy repulsion model, and relaxations around vacancies are characterized.

## 1. Introduction   

Crystalline solids are typically understood as being periodic on the atomic scale, and are described by a repeating unit cell in three dimensions. With such an ordered arrangement of atoms, knowledge of the atomic configuration in one part of the crystal will perfectly predict the positions of all other atoms. This is in contrast to amorphous solids, where there are only local coordination rules. Knowledge of the atomic configuration at one point in an amorphous solid does not allow prediction of atomic positions elsewhere in the material.

In general, the atomic structure of a material determines its properties. In crystals, collective behaviour of the periodic structures gives rise to delocalized modes in the electronic, magnetic and vibrational properties. For amorphous materials, the properties arise from localized behaviour. Between the two extremes we find disordered crystals, which on average can be described by an ordered periodic structure, but with deviations on a local scale. These can have more complex properties arising from both collective and localized behaviour. The average periodic structure gives rise to sharp peaks in a diffraction experiment (Bragg peaks), which can be readily analysed. The local deviations give rise to weak diffuse scattering, which is more difficult to measure and especially to interpret on a structural basis (Keen & Goodwin, 2015 ▸; Krogstad *et al.*, 2020 ▸; Simonov *et al.*, 2020 ▸).

Defective half-Heusler materials *X*_1−*x*_ *YZ*, such as Nb_1−*x*_CoSb, have excellent thermoelectric properties and show signs of local atomic order different from their average periodic order (Anand *et al.*, 2018 ▸; Huang *et al.*, 2015 ▸; Xia *et al.*, 2018 ▸; Zeier *et al.*, 2017 ▸; Zhang *et al.*, 2016 ▸). Electron diffraction data have revealed structured diffuse scattering, which differ from sample to sample (Xia *et al.*, 2019 ▸). The differences were first explained as a result of different nominal sample stoichiometry (Xia *et al.*, 2019 ▸), but recently the differences in shape of the reported diffuse scattering have been theoretically explained by a simple model for effective vacancy repulsion on the disordered *X* substructure, without invoking different sample stoichiometry (Roth *et al.*, 2020 ▸). The model suggests that the structure places vacancies as far apart as possible, essentially giving the effect of vacancy repulsion. Importantly, the model predicts that the degree of local order can be influenced by synthesis conditions, such as thermal quenching. Experimental tuning of local order would provide a new handle in materials research, which may allow for controlling properties in disordered systems.

Recently, Simonov and coworkers used diffuse X-ray scattering data to establish vacancy distributions in Prussian Blue Analogue (PBA) materials (Simonov *et al.*, 2020 ▸). Different PBAs crystals showed dissimilar diffuse scattering patterns, and indeed PBA materials are known to have large variation, for example in battery properties (Kjeldgaard *et al.*, 2021 ▸). However, an understanding of how local structure can be controlled and how the local structure correlates with material properties is still largely unexplored. Here, we show that the synthesis conditions change the degree of local order in thermoelectric Nb_1−*x*_CoSb. Using diffuse synchrotron X-ray scattering data measured on single crystals, we first validate the theoretical vacancy repulsion model, and then we model the structural relaxation around the vacancies to provide direct experimental quantification of the local structure in these systems.

## 2. Experimental   

### 2.1. Synthesis   

Two types of synthesess were used to produce samples with nominal stoichiometries Nb_0.81_CoSb and Nb_0.84_CoSb. One group of samples was thermally quenched from the melt (levitation-melt technique), and the other group of samples was slowly cooled using an induction furnace. The quenched (Q) samples are of the same type used in the published electron diffraction study by Xia *et al.* (2019 ▸). Slowly cooled (SC) samples of compositions Nb_0.81_CoSb and Nb_0.84_CoSb were prepared from stoichiometric mixtures of the pure elements with a 1% molar excess of Sb to compensate for its evaporation during synthesis. Pieces of Sb and Co, and wire clippings of Nb were loaded into an alumina crucible, which was then slotted into a graphite susceptor inside an induction coil. The furnace chamber was evacuated thrice to a level between 0.01 and 0.1 mbar with a back filling of He between evacuations, before being pressurized to 10 bar with He. The output power of the furnace was turned up in large steps over a 5 min period to the point where a homogeneous melt is achieved. Based on the light emitted from the susceptor this occurs between 1400 and 1500°C. After a 1 min soak time at the maximum temperature, the output power was gradually decreased over 5 min until the whole melt had solidified, at which point the temperature of the susceptor was around 1100°C. After the sample had solidified, the furnace was turned down and finally off over a period of 5 min, after which the sample was left in the furnace chamber for 10 min to cool to room temperature. Comparing the mass of the materials used and the samples obtained shows that only between 0.015 and 0.02 g are lost during synthesis of a 10 g sample, which corresponds to only between 1/3 and 1/2 of the added excess Sb.

### 2.2. X-ray scattering measurements   

Data were collected on five samples: Q Nb_0.81_CoSb (Q-0.81), SC Nb_0.81_CoSb (SC-0.81), two Q Nb_0.84_CoSb samples (Q-0.84#1 and Q-0.84#2) and one SC Nb_0.84_CoSb (SC-0.84). Single crystals 50–80 µm in size were glued to the end of thin glass pins using ep­oxy and mounted on a goniometer. Data were measured at the BL02B1 beamline at the SPring-8 synchrotron using a photon energy of 50.00 keV on a Huber four-circle (quarter chi) goniometer equipped with a Pilatus3 X 1M CdTe (P3) detector. For the Q samples a detector distance of 130 mm was used, whereas 260 mm was used for the SC samples to properly separate the sharp additional peaks.

Two measurements were made for each sample. First, a dataset for the strongest reflections was collected. For this, a 600 µm Ni film was used to attenuate the beam to 31% to avoid problems of too high flux on the detector. Here four runs were measured, each a 180° ω rotation with 900 frames, for χ = 0 and χ = 45° with the detector at 2θ = 0 and 2θ = −25°. An exposure time of 0.8 s per frame was used. Then the weak scattering was measured without any beam attenuator. Here six runs were measured, each a 180° ω rotation with 900 frames, χ = 0 and χ = 45° with the detector at 2θ = 0, 2θ = −12.5 and 2θ = −25°. An exposure time of 1.6 s per frame was used. Finally the background and air-scattering were measured using the same set of exposure times, detector positions and beam attenuation as for the crystal measurement. For each combination, 200 frames of air scattering were measured.

To obtain the average structure, the images were converted to the Bruker .sfrm format (Krause *et al.*, 2020 ▸) and the Bragg peaks were integrated using *SAINT* (Bruker, 2013 ▸). The integrated data were processed and corrected using *SADABS* (Krause *et al.*, 2015 ▸) and merged using *SORTAV* (Blessing, 1997 ▸). Initial structure solution and refinement was carried out with *SHELXS* and *SHELXL* using the *Olex2* GUI (Dolomanov *et al.*, 2009 ▸; Sheldrick, 2008 ▸), with subsequent structure refinement using *JANA2006* (Petříček *et al.*, 2014 ▸). The space group is 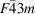 . Anomalous scattering factors for 50 keV were used, as implemented in *JANA2006*.

For the diffuse scattering analysis the data were converted to reciprocal space using a custom Matlab script. During this process the data were corrected for polarization, the background scattering from air was subtracted and a solid-angle correction was applied. The resulting data were symmetrized using the 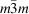 point symmetry of the Laue group. The resulting scattering data were reconstructed on a 901 × 901 × 901 point grid with each axis spanning ±27 Å^−1^ for the Q samples and ±21 Å^−1^ for the SC furnace samples.

For the production of a 3D-ΔPDF, the Bragg peaks of the average structure were punched and filled. Because the Bragg peaks and diffuse scattering do not overlap, the Bragg peaks were removed using a spherical punch based on the positions of allowed reflections of 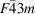 . The punched holes were then filled using a 3D spline interpolation. Outside the area of measured data, a constant value was added to match the edges of the measured regions, as to avoid strong Fourier ripples. The resulting data containing only the diffuse scattering/additional peaks were then Fourier transformed to give the 3D-ΔPDF.

### 2.3. Simulated data   

Monte–Carlo simulations of the structure were performed using the Metropolis algorithm (Metropolis *et al.*, 1953 ▸) using a custom python script. The model scattering was calculated using the *Scatty* software (Paddison, 2019 ▸) and the model 3D-ΔPDF was obtained by Fourier transforming the model scattering using a custom python script.

## 3. Results   

The theoretical model for vacancy repulsion in defective half-Heusler materials predicts that samples quenched from high temperature will have a different degree of local order than SC samples (Roth *et al.*, 2020 ▸). Using induction furnaces, two distinct types of samples have been synthesized, Q samples from a levitation-melt procedure and SC samples using crucibles. Samples were prepared for both methods with nominal stoichiometries of Nb_0.81_CoSb and Nb_0.84_CoSb, and they are named such that ‘Q-0.81’ is a Q sample with nominal stoichiometry Nb_0.81_CoSb.

From very high-quality single-crystal X-ray scattering measurements performed at a synchrotron, we obtained the average crystal structure for each single crystal from the Bragg peaks, including an accurate stoichiometry. In addition, from the same measurement we obtain the diffuse scattering, allowing for analysis of the local order. With this method we can directly correlate the diffuse scattering and local order with the periodic crystal structure and sample composition, as this is all obtained from one measurement for each single crystal. It further avoids errors in determining the sample composition using other methods, as impurity phases are known to occur in these compounds (Xia *et al.*, 2019 ▸).

### 3.1. Average crystal structure   

**Figure 1:**
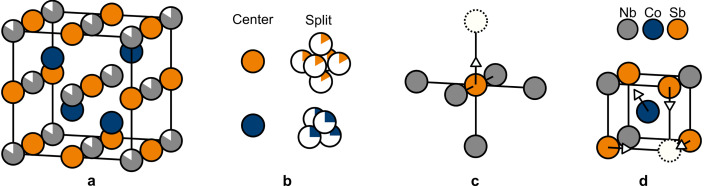
Crystal Structure of Nb_1−*x*_CoSb. (*a*) Unit cell of the average structure. Blue: Co, grey; Nb, orange: Sb. Partial shading indicates occupancy. (*b*) Two models have been tested, one with Sb and Co at the ideal half-Heusler positions and one with off-centred Sb and Co (exaggerated in the figure). (*c*) Nb_5_Sb elementary block with the Sb displacement indicated by the arrow. (*d*) Local relaxation of Co close to a vacancy with the movements of Sb and Co indicated by arrows.

The average periodic crystal structures in all samples were quantified by analysis of the Bragg diffraction peaks. All samples have cubic average structures in the space group 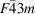 with the cell lengths in a narrow range between 5.894 and 5.899 Å at 300 K. The quality of the Bragg data is excellent with internal agreement (*R*_int_) between 4.0 to 5.4% even for high data resolutions with *d*_min_ < 0.43 Å, and average redundancies of 19–36 (see Table 1 ▸). Previous studies describe the average structure as an ideal half-Heusler structure with vacancies on the Nb sites (Zeier *et al.*, 2017 ▸). The ideal half-Heusler structure (space group 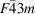 ) has Nb_1−*x*_ at [0, 0, 0], Sb at [1/2, 1/2, 1/2] and Co at [1/4, 1/4, 3/4]. Nb_1−*x*_ and Sb form a rock-salt structure, whereas Nb and Co form the sphalerite structure, as illustrated in Fig. 1 ▸(*a*).

Refining a model with free Nb occupancy gives good (low) agreement factors (*R*_1_: 2.29–3.23%, *wR*_2_: 5.94–7.39%), which would normally be considered an excellent fit, especially with the large range of data. However, clear residuals are observed around the Sb and Co sites suggesting disorder, as shown in detail in the supporting information. If Sb is positioned off-centre at [1/2+Δ, 1/2, 1/2] with refinement of Δ, and Co is off-centred at [1/4-δ, 1/4-δ, 3/4-δ] with refinement of δ, the agreement factors improve significantly for all samples (*R*_1_: 0.49–1.06%, *wR*_2_: 0.78–1.33%), Table 1 ▸. In this model, each Sb is split into six positions (each with 1/6 occupancy) forming an octahedron with corners pointing towards the six neighbouring Nb_1−*x*_ sites, while Co is split into a tetrahedron with corners pointing towards neighbouring Sb, see Fig. 1 ▸(*b*). For all samples, the Sb shift refines to Δ ≃ 0.025 corresponding to a movement of 0.144 Å, and Co shifts δ ≃ 0.0127 corresponding to a movement of 0.130 Å. Interestingly, the refined Nb occupancies are in the narrow range 0.820–0.827 for the five samples, suggesting that all Nb_1−*x*_CoSb samples are very close in stoichiometry with *x* ≃ 1/6. Thus, there is no correlation between the nominal and refined sample stoichiometry. From refinements of the Bragg data all samples are identical, but ‘hidden’ local structures are exposed through analysis of the diffuse scattering.

### 3.2. Tuneable short-range order   

**Figure 2:**
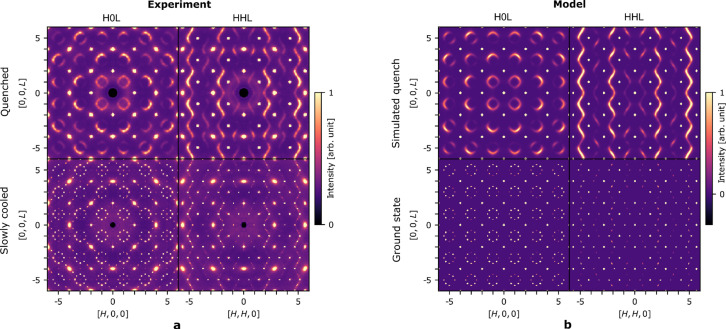
Measured and calculated X-ray scattering in the H0L and HHL planes. (*a*) Measured X-ray scattering from two representative samples of Nb_1−*x*_CoSb at 300 K with the top row being a Q sample and the bottom row an SC sample. (*b*) Calculated X-ray scattering with the top row showing a simulated quench model, and the bottom showing a ground state of type ‘BD’ (see text).

Although all samples have almost identical average structures, their scattering patterns are highly different. In general, the thermally quenched (Q) samples show more diffuse scattering than the SC samples, which have sharp additional peaks. Fig. 2 ▸(*a*) shows the measured scattering in the H0L and HHL planes for two representative samples, and scattering patterns from the remaining samples are provided in the supporting information. There is no correlation between the nominal sample stoichiometry and the degree of diffuse scattering, but clearly the diffuse scattering depends on the synthesis method. The inverse of the broadness of the diffuse scattering is proportional to the correlation length of local order. Thus, the Q samples with broader diffuse scattering only have short-range order, whereas the SC samples with sharp peaks have longer range order.

The measured X-ray scattering data [Fig. 2 ▸(*a*)] differ significantly from the electron diffraction data reported by Xia *et al.* (2019 ▸), where the diffuse scattering consists of rings in the H0L plane with approximately constant intensity around the perimeter. In the X-ray data, the rings have clear intensity modulations. The difference can be attributed to strong multiple scattering in the electron diffraction data, where the electron beam of strong Bragg peaks is re-scattered to give diffuse scattering averaged over Brillouin zones. This creates rings of approximately constant intensity, especially when data are measured along the zone-axis where Bragg scattering is strong, which is the case for the data in Xia *et al.* (2019 ▸)

The theoretical model for the vacancy distribution (Roth *et al.*, 2020 ▸) was inspired by the electron diffraction data, and it produces calculated rings without the intensity modulation observed in the present X-ray scattering data. As will be shown, the intensity modulation of the diffuse scattering is mainly the result of structural relaxation of Sb and Co around vacancies, but overall the vacancy ordering model is validated. Similar modulations due to structural relaxation around vacancies, a type of size-effect, have been reported in other compounds (Oeckler *et al.*, 2005 ▸; Welberry, 1986 ▸).

To get a direct view of the local correlations in the samples, the diffuse scattering is Fourier transformed to obtain the thee-dimensional difference pair distribution function (3D-ΔPDF), which is the autocorrelation of the difference electron density:

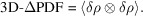

Here 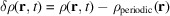 is the difference between the total electron density of the crystal and the periodic average electron density. The 3D-ΔPDF gives a direct view of the local deviations from the periodic average structure (Canut-Amorós, 1967 ▸; Schaub *et al.*, 2007 ▸; Weber & Simonov, 2012 ▸). Positive/negative features show vectors for which the real structure has more/less electron density separated by those vectors compared with the average structure. The types and signs of features can be directly related to the types of local correlations (Weber & Simonov, 2012 ▸), and indeed the 3D-ΔPDF has been used to solve the local order in several disordered crystals (Krogstad *et al.*, 2020 ▸; Roth & Iversen, 2019 ▸; Sangiorgio *et al.*, 2018 ▸).

**Figure 3:**
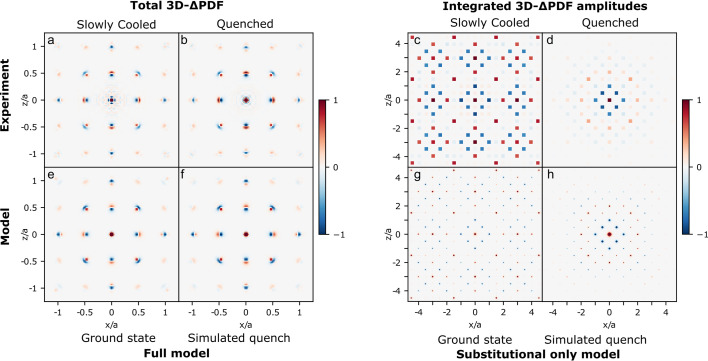
Measured and calculated 3D-ΔPDF. (*a*) and (*b*) Measured 3D-ΔPDF for the ‘SC-0.81’ and ‘Q-0.84 #2’ samples in the 010 plane (same samples as in Fig. 2 ▸). (*c*) and (*d*) Integrated peak amplitudes of the 3D-ΔPDF. (*e*) and (*f*) Calculated 3D-ΔPDF using the vacancy repulsion model *including* Sb and Co relaxation. Both plots have a vacancy concentration of *x* = 1/6 but (*e*) represents a BD-type ground state and (*f*) a simulated quench with *J*_2_/*J*_1_ = 0.5. Details of the ground-state structure are given in the supporting information. (*g*) and (*h*) Calculated 3D-ΔPDF using the vacancy repulsion model without Sb and Co displacements.

Figs. 3 ▸(*a*) and 3(*b*) show the 3D-ΔPDF in the 010 plane. The short-range features are almost identical in the two representative samples, but the features decay quickly in the Q sample while they have longer range for the SC sample. This is illustrated in more detail in the supporting information. In general, the 010 plane is dominated by features which are positive on one side and negative on the other, a direct indication of strong local relaxation around vacancies (Weber & Simonov, 2012 ▸).

### 3.3. Vacancy ordering   

To remove the effect of atomic movements and isolate the effect of local vacancy ordering, the features in the 3D-ΔPDF can be integrated. As shown by Roth & Iversen (2019 ▸), the integral amplitudes of features in the 3D-ΔPDF relate only to the substitutional disorder. The integrated peak amplitude of a peak at position **r**′ is proportional to

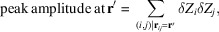

where the summation runs over all pairs of atoms (*i*, *j*) separated by vector **r** *_ij_* = **r**′, and δ*Z*_*i*_ is the difference in the number of electrons of atom *i* in the real structure compared with the average periodic structure. It is possible to use this method on the current samples as the features in the 3D-ΔPDF are well separated. Details of the peak integration are given in the supporting information.

The integral peak amplitudes are shown in Figs. 3 ▸(*c*) and 3(*d*). A positive value is found at the origin since atoms are separated by the zero vector to themselves. Surrounding the origin are strong negative integral amplitudes corresponding to the nearest and next-nearest vectors in the Nb substructure [coordinates (1/2,1/2,0) and (1,0,0)]. This shows that there is a lower-than-average probability of finding two atoms separated by these vectors. This means that the structure tends to avoid the nearest and next-nearest vacancy pairs. Then at slightly longer distances there are several positive amplitudes showing the preferred distances between the vacancies. Again, the SC samples have strong correlations to much longer distances than the Q samples.

The integral amplitudes can then be directly compared with the calculated 3D-ΔPDF obtained from a Monte–Carlo simulation of the vacancy distribution using the vacancy repulsion model (Roth *et al.*, 2020 ▸). The model gives a positive energy to each nearest and next-nearest vacancy pair with the total energy of the system given by

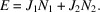

Here, *N*_1_ is the number of nearest-neighbour vacancy pairs and *N*_2_ is the number of next-nearest-neighbour vacancy pairs on the Nb substructure. *J*_1_ and *J*_2_ are the energy penalties of the nearest and next-nearest neighbour vacancy pairs. The vacancy concentration *x* = 1/6 is the highest vacancy concentration where it is possible to avoid all nearest and next-nearest neighbour vacancy pairs, as shown by Roth *et al.* (2020 ▸). The ground states for the model are therefore all the configurations with no such pairs (*E* = 0). There is a large number of configurations satisfying those rules, and different possible types were previously identified (Roth *et al.*, 2020 ▸). The integrated amplitudes for the SC samples agree with the calculated 3D-ΔPDF for a vacancy model in the ground state, as shown in Fig. 3 ▸(*g*). The amplitudes are in best agreement with the ground-state type ‘BD’ (details in the supporting information).

For the non-ground-state configurations, a Monte–Carlo simulation can be carried out using the Metropolis algorithm (Metropolis *et al.*, 1953 ▸). Steps with a positive energy change are accepted with the probability 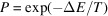 , where *T* is the simulated temperature. To simulate the Q samples, a configuration with *x* = 1/6 is started from random vacancy positions, and the simulation is run for *T* = 0. The relative energy penalty for next-nearest vacancy pairs was set to half the energy penalty of the nearest pairs, *J*_2_/*J*_1_ = 1/2. The resulting simulated 3D-ΔPDF is in good agreement with the integrated amplitudes from the Q samples, as shown in Fig. 3 ▸(*h*).

For both the Q and SC samples, the vacancy correlations from the simulation correspond well with those obtained by integration of amplitudes in the measured 3D-ΔPDF. This shows that the model for vacancy correlations (Roth *et al.*, 2020 ▸) agrees with the experimental data, even though the model does not reproduce the intensity modulations of the diffuse scattering rings.

### 3.4. Structural relaxation around vacancies   

The measured 3D-ΔPDF [Figs. 3 ▸(*a*) and 3(*b*)] show strong features from local structural relaxation around vacancies. At (*x*,*y*,*z*) = (1/2,0,0), the 3D-ΔPDF is negative towards the centre and positive away from the centre. This is the vector between Nb/vacancies and Sb. This means that when a Nb is present, Sb will be slightly further away, and when a vacancy is present, Sb will move towards the vacancy position.

In the average structure each Sb is surrounded by six close Nb/vacancy positions, forming an octahedron. Avoidance of the nearest and next-nearest vacancy requires each such octahedron to have at most one vacancy out of six corners. For *x* = 1/6 there will be one and only one close vacancy per Sb in the ground state. This means that each Sb simply moves towards the one vacancy neighbour it has by approximately 0.144 Å. Consequently, the (Nb,Sb) substructure can be seen as being built from Nb_5_Sb square pyramidal units, where Sb is displaced slightly towards the empty Nb site, as illustrated in Fig. 1 ▸(*c*).

**Figure 4:**
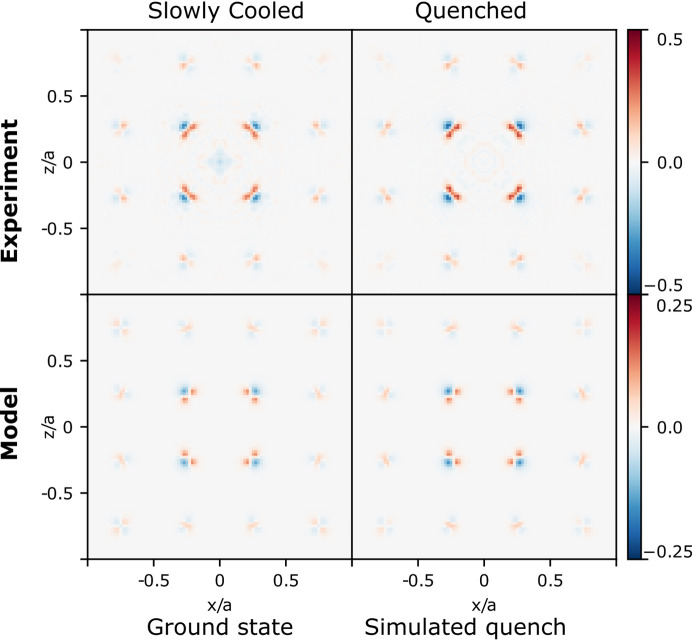
Measured and calculated 3D-ΔPDF in the *y* = 0.27*a* plane. This plane shows vectors which are all related to correlations between Co and other atoms. The top row shows the measured 3D-ΔPDF for the two representative samples, and the bottom row shows the corresponding 3D-ΔPDF calculated from the vacancy repulsion model with structural relaxations. Note that the experiment and model 3D-ΔPDF are shown on different scales. The measured 3D-ΔPDF has stronger amplitudes, indicating the model to still have something missing.

The relaxation of Co close to vacancies can also be identified from the 3D-ΔPDF. The top row in Fig. 4 ▸ shows the (*x*, *y* = 0.27*a*, *z*) plane of the 3D-ΔPDF for the two representative samples. This plane shows strong features which are all related to correlations between Co and the other atoms. Each Co sits in a cube formed by Sb and Nb_1−*x*_. The shortest Co–Nb and Co–Sb vectors, which are identical in the average structure, are located around (0.25, 0.25, 0.25). As seen in the top of Fig. 4 ▸, the feature is mostly positive towards the origin and negative away from the origin. This means Co moves away from a vacant Nb site, consistent with the tetrahedron of Co sites in the average structure. This is illustrated in Fig. 1 ▸(*d*), where the local environment of Co close to a vacancy is shown. Since the local relaxation of both Sb and Co has been identified from both the average structure and the 3D-ΔPDF, the local structure model can be improved and compared with the measurements.

To improve the theoretical model, the vacancy distributions obtained from the Monte–Carlo simulations are used, but with each Sb moved by 0.144 Å toward its neighbouring vacancy, and with Co moved by 0.130 Å away from neighbouring vacancies. The calculated 3D-ΔPDFs for this model are shown in Figs. 3 ▸(*e*) and 3(*f*) for the (010) plane. These are in good agreement with the experimentally obtained 3D-ΔPDF maps, further validating the local structural relaxation around the vacancies. The bottom row of Fig. 4 ▸ shows the calculated 3D-ΔPDF in the *y* = 0.27*a* plane and a good agreement with experiment is observed. However, the amplitudes of the features are significantly weaker for the model than for the experiment, suggesting still undescribed features in the model. These could be small relaxations of Nb in its local environment, or possibly more complex movements of Co/Sb than investigated here.

The calculated scattering for the models is shown in Fig. 2 ▸(*b*). The intensity modulations of the diffuse scattering are now, to a large extent, reproduced by the model. Both the short-range order of the distribution of Nb/vacancies and the displacements of Sb and Co are needed to explain the measured data. A previous study erroneously claimed that the Nb/vacancy ordering was insignificant to the diffuse scattering signal (Nan *et al.*, 2020 ▸), as explained in the supporting information. There are still small differences in the measured and calculated scattering. For example, there are weak additional peaks for the SC samples not currently reproduced by the model. This is discussed in the supporting information.

## 4. Discussion   

The Q samples show more diffuse-like scattering than the SC samples, which have sharp additional peaks. This shows that the degree of local order can be tuned through the synthesis conditions (Roth *et al.*, 2020 ▸). Surprisingly, the refined chemical compositions are virtually identical in all samples, *x* ≃ 1/6, even though the nominal stoichiometry used in the syntheses differ substantially. Accurate determination of the composition was made possible by the high-quality single-crystal X-ray scattering data, avoiding errors from other impurity phases known to occur in these systems (Xia *et al.*, 2019 ▸). This suggests that the stable composition is Nb_5/6_CoSb, and that there is very little room for variation in composition. Previous studies have argued that the ideal composition is Nb_4/5_CoSb based on simple valence electron counting (Zintl) rules (Zeier *et al.*, 2017 ▸). However, *x* = 1/6 is the highest vacancy concentration for which both the nearest and next-nearest vacancy pairs can be avoided completely (Roth *et al.*, 2020 ▸). This means that the energetic penalty for disturbing the local chemical bonding in the locally ordered vacancy structure is higher than the presumed gain in electronic energy expected from simplistic electron counting. It is noteworthy that the average structure is identical for all five samples, and also that the local structural relaxation is the same with Sb being off-centred by 0.144 Å and Co off-centred by 0.130 Å. Each Sb has one vacant neighbour and moves toward it, while Co moves away from neighbouring vacancies.

Having established that all samples have *x* ≃ 1/6, but differ in their local structure, a key question is how the local structure affects the thermoelectric properties. It is difficult to measure thermoelectric properties on samples with controlled local structure, since fabrication of a pellet from polycrystalline material requires densification under heat and pressure. However, Xia *et al.* reported the thermoelectric properties of six samples with *x* varying between 0.15 and 0.20, all synthesized by the quench method. The samples had identical lattice parameters (from powder X-ray diffraction) strongly corroborating that *x* = 1/6 for all samples as suggested here. The differences in carrier concentrations are likely affected by the necessary impurities present when nominal compositions deviate from 1/6. The measured transport data strongly reflect the presence of vacancies in their temperature behaviour, but presumably it is the lattice thermal conductivity, κ_L_, which is most directly affected by the local structure. At room temperature, the samples differ by about 15% in κ_L_ and this could indeed be a direct effect of different vacancy distributions. It could therefore be advantageous to use these differences in properties. Electron diffraction data showed the diffuse features to be stable even at 1000 K, suggesting the vacancy distributions to be stable to quite high temperatures (Xia *et al.*, 2019 ▸), making it possible to produce devices with a specific vacancy order.

## 5. Conclusions   

In summary, we have shown that defective half-Heusler materials (Nb_1−*x*_CoSb) have a strong tendency to vacancy ordering following a simple repulsion model. The optimal ordering of vacancies essentially fixes the stoichiometry of the samples irrespective of the nominal starting composition or synthesis method. Using analysis of both Bragg diffraction and diffuse scattering data with the 3D-ΔPDF method, it is possible to quantify the structural relaxation around a vacancy site. Different local-structure states are reached depending on the thermal treatment of the sample, and they appear to have appreciable effect on the thermoelectric properties. Advanced X-ray scattering techniques can unravel hidden local structures and for Nb_1−*x*_CoSb these local structures can be controlled by the synthesis conditions. If the local structure of crystalline materials can be more generally related to the properties, then a new frontier in materials research will be available.

## Supplementary Material

Supporting figures. DOI: 10.1107/S2052252521005479/fc5055sup1.pdf

## Figures and Tables

**Table 1:** Average structure refinements for the different Nb_1−*x*_CoSb samples Q: quenched samples, SC: slowly cooled samples. The decimal number designates the nominal Nb stoichiometry. Avg. red. is the average data redundancy, *i.e.* the average number of times each unique reflection was measured.

					Center model			Split model				
Sample	*a* (Å)	Avg. red.	*d*_min_ (Å)	*R*_int_ (%)	*R*_1_ (%)	*wR*_2_ (%)	occ_Nb_	*R*_1_ (%)	*wR*_2_ (%)	occ_Nb_	Sb shift (Å)	Co shift (Å)
Q-0.81	5.896	33	0.33	4.32	2.27	6.06	0.827	0.63	0.83	0.827	0.142	0.131
SC-0.81	5.894	34	0.43	4.02	2.28	6.20	0.835	0.89	1.16	0.820	0.148	0.128
Q-0.84 #1	5.899	19	0.40	5.36	3.23	7.39	0.820	1.06	1.33	0.825	0.142	0.130
Q-0.84 #2	5.896	36	0.40	4.28	2.76	6.00	0.831	0.49	0.78	0.827	0.141	0.130
SC-0.84	5.895	29	0.40	4.16	2.94	5.94	0.820	0.98	1.27	0.822	0.146	0.131
Average	5.896	30	0.39	4.43	2.70	6.32	0.827	0.81	1.07	0.824	0.144	0.130

## References

[bb1] Anand, S., Xia, K., Zhu, T., Wolverton, C. & Snyder, G. J. (2018). *Adv. Energy Mater.* **8**, 1801409.

[bb2] Blessing, R. H. (1997). *J. Appl. Cryst.* **30**, 421–426.

[bb3] BRUKER (2013). *Saint - plus integration engine..*

[bb4] Canut-Amorós, M. (1967). *Z. Kristallogr.* **124**, 241–261.

[bb5] Dolomanov, O. V., Bourhis, L. J., Gildea, R. J., Howard, J. A. K. & Puschmann, H. (2009). *J. Appl. Cryst.* **42**, 339–341.

[bb6] Huang, L., He, R., Chen, S., Zhang, H., Dahal, K., Zhou, H., Wang, H., Zhang, Q. & Ren, Z. (2015). *Mater. Res. Bull.* **70**, 773–778.

[bb7] Keen, D. A. & Goodwin, A. L. (2015). *Nature*, **521**, 303–309. 10.1038/nature14453 25993960

[bb8] Kjeldgaard, S., Dugulan, I., Mamakhel, A., Wagemaker, M., Iversen, B. B. & Bentien, A. (2021). *R. Soc. Open Sci.* **8**, 201779. 10.1098/rsos.201779 PMC7890497 33614096

[bb9] Krause, L., Herbst-Irmer, R., Sheldrick, G. M. & Stalke, D. (2015). *J. Appl. Cryst.* **48**, 3–10. 10.1107/S1600576714022985 PMC4453166 26089746

[bb10] Krause, L., Tolborg, K., Grønbech, T. B. E., Sugimoto, K., Iversen, B. B. & Overgaard, J. (2020). *J. Appl. Cryst.* **53**, 635–649. 10.1107/S1600576720003775 PMC7312157 32684879

[bb11] Krogstad, M. J., Rosenkranz, S., Wozniak, J. M., Jennings, G., Ruff, J. P. C., Vaughey, J. T. & Osborn, R. (2020). *Nat. Mater.* **19**, 63–68. 10.1038/s41563-019-0500-7 31636421

[bb12] Metropolis, N., Rosenbluth, A. W., Rosenbluth, M. N., Teller, A. H. & Teller, E. (1953). *J. Chem. Phys.* **21**, 1087–1092.

[bb13] Nan, P., Wu, K., Liu, Y., Xia, K., Zhu, T., Lin, F., He, J. & Ge, B. (2020). *Nanoscale* **12**, 21624–21628. 10.1039/d0nr04957c 32756706

[bb14] Oeckler, O., Weber, T., Kienle, L., Mattausch, H. & Simon, A. (2005). *Angew. Chem. Int. Ed.* **44**, 3917–3921. 10.1002/anie.200500594 15892127

[bb15] Paddison, J. A. M. (2019). *Acta Cryst.* A**75**, 14–24. 10.1107/S2053273318015632 30575580

[bb16] Petříček, V., Dušek, M. & Palatinus, L. (2014). *Z. Kristallogr.* **229**, 345–352.

[bb17] Roth, N. & Iversen, B. B. (2019). *Acta Cryst.* A**75**, 465–473. 10.1107/S2053273319004820 31041902

[bb18] Roth, N., Zhu, T. & Iversen, B. B. (2020). *IUCrJ*, **7**, 673–680. 10.1107/S2052252520005977 PMC7340261 32695414

[bb19] Sangiorgio, B., Bozin, E. S., Malliakas, C. D., Fechner, M., Simonov, A., Kanatzidis, M. G., Billinge, S. J. L., Spaldin, N. A. & Weber, T. (2018). *Phys. Rev. Mater.* **2**, 085402.

[bb20] Schaub, P., Weber, T. & Steurer, W. (2007). *Philos. Mag.* **87**, 2781–2787.

[bb21] Sheldrick, G. M. (2008). *Acta Cryst.* A**64**, 112–122. 10.1107/S0108767307043930 18156677

[bb22] Simonov, A., De Baerdemaeker, T., Boström, H. L. B., Ríos Gómez, M. L., Gray, H. J., Chernyshov, D., Bosak, A., Bürgi, H.-B. & Goodwin, A. L. (2020). *Nature*, **578**, 256–260. 10.1038/s41586-020-1980-y PMC7025896 32051599

[bb23] Weber, T. & Simonov, A. (2012). *Z. Kristallogr.* **227**, 238–247.

[bb24] Welberry, T. R. (1986). *J. Appl. Cryst.* **19**, 382–389.

[bb25] Xia, K., Liu, Y., Anand, S., Snyder, G. J., Xin, J., Yu, J., Zhao, X. & Zhu, T. (2018). *Adv. Funct. Mater.* **28**, 1705845.

[bb26] Xia, K., Nan, P., Tan, S., Wang, Y., Ge, B., Zhang, W., Anand, S., Zhao, X., Snyder, G. J. & Zhu, T. (2019). *Energy Environ. Sci.* **12**, 1568–1574.

[bb27] Zeier, W. G., Anand, S., Huang, L., He, R., Zhang, H., Ren, Z., Wolverton, C. & Snyder, G. J. (2017). *Chem. Mater.* **29**, 1210–1217.

[bb28] Zhang, H., Wang, Y., Huang, L., Chen, S., Dahal, H., Wang, D. & Ren, Z. (2016). *J. Alloys Compd.* **654**, 321–326.

