# Supplementary material for: Tuneable local order in thermoelectric crystals
Source: IUCrJ. 2021 Jun 30;8(Pt 4):695–702. doi: 10.1107/S2052252521005479 (PMC8256708; doi:10.1107/S2052252521005479)
Supplement: Supplementary file 1 [file m-08-00695-sup1.pdf]

IUCrJ

Volume 8 (2021)

Supporting information for article:

Tuneable local order in thermoelectric crystals

Nikolaj Roth, Jonas Beyer, Karl F. F. Fischer, Kaiyang Xia, Tiejun Zhu and Bo B. Iversen

S1. Residual density in the average structure refinements

Figure S1 shows the residual electron density, calculated as an F_{obs}-F_{calc} Fourier map for the two models of the average structure. Both the [x,0.5,z] and [x,-x,z] planes are shown. The upper row illustrates the positons of atoms in these planes. There are clear residuals around Sb and Co for the center model, showing the models to be incomplete. Because of the incomplete description, there are also spurious features at positions with no atoms in the structure, e.g. in the [x,-x,z] plane around [0.25,-0.25,0.75]. After refinement with the split site model, the residuals disappear, including the spurious features, leaving a flat and featureless difference map.

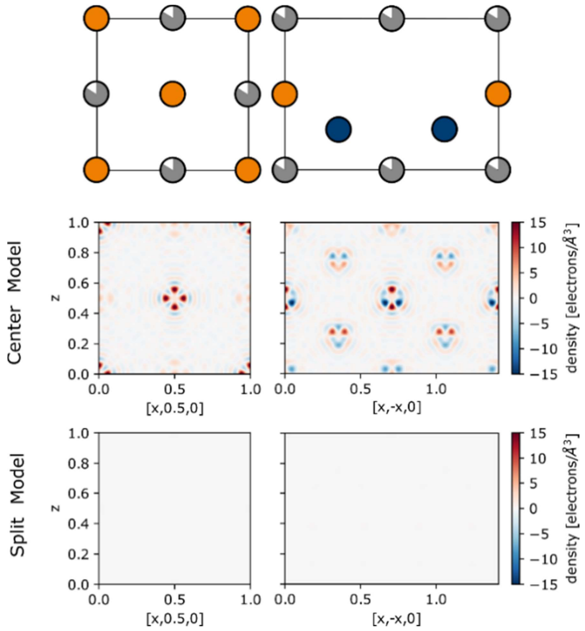

Figure S1 Residual electron density after refinement of the two models for the average structure. The left column shows the [x,0.5,z] plane, while the right column shows the [x,-x,z] plane. The upper row shows the ideal half-Heusler positions of atoms in these two planes. The middle row shows the residual electron density for the center model, while the bottom row is for the split model.

S2. Measured X-ray scattering data

The measured X-ray scattering in the H0L and HHL planes are shown below for all samples.

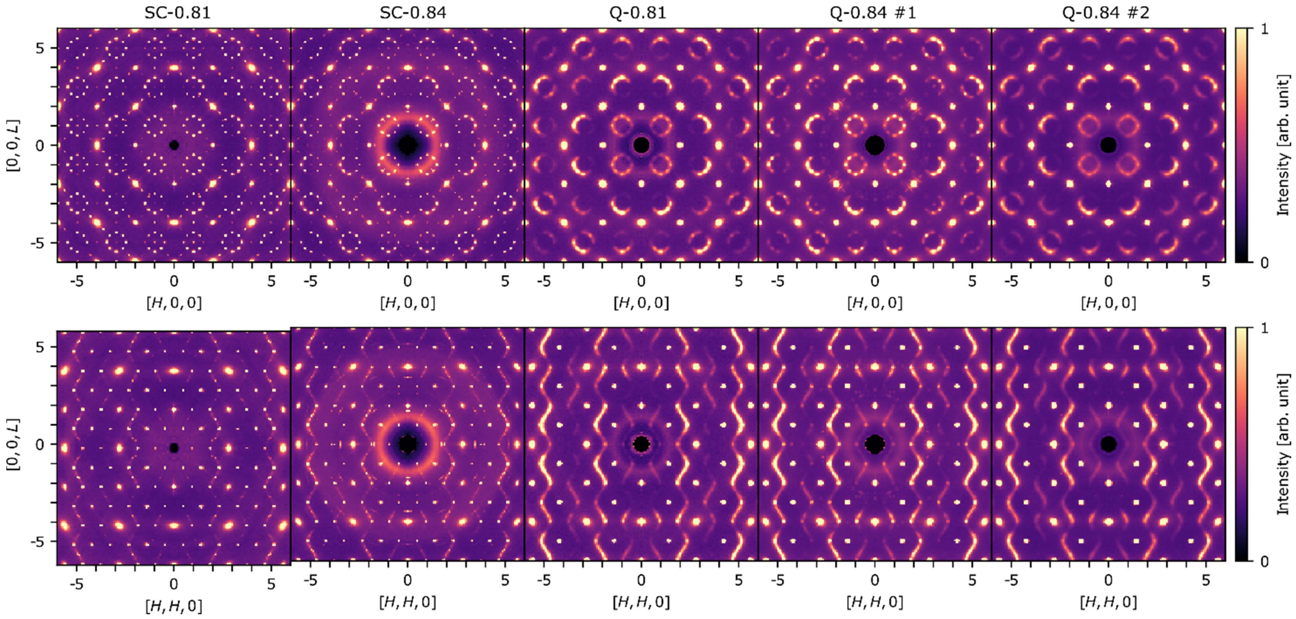

Figure S2 Measured X-ray scattering in the H0L and HHL plans for the different samples. For the levitation-melt synthesis with nominal stoichiometry $Nb_{0.84}CoSb$, data were measured on two crystals ("Q-0.84 # 1" and "Q-0.84 \# 2"). While crystal "Q-0.84 \# 2" shows quite broad diffuse scattering, crystal "Q-0.84 # 1" has sharper additional peaks. This suggests that the levitation-melt samples have inhomogeneities in the degree of short-range order, which might occur as different parts of the sample are cooled at different rates during the quenching process.

S3. Integrated peak amplitudes

Peak integration is done by summing all voxels of the 3D-ΔPDF in a three-dimensional box around each feature. The used boxes are illustrated in Figure S3. Here the "Q-0.84 #1" sample is used to illustrate the method.

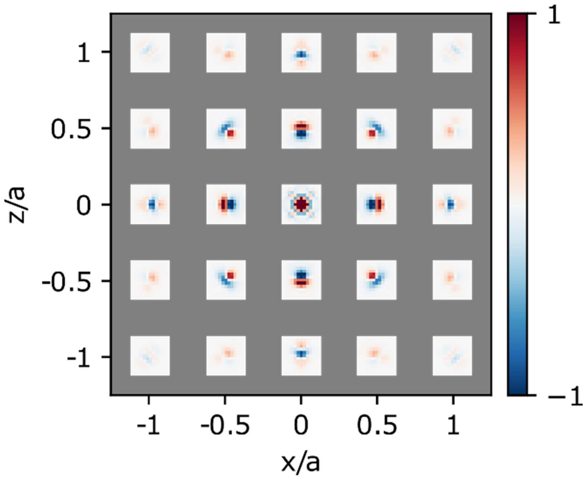

Figure S3 Two-dimensional view of the 3D integration boxes around features.

S4. Longer data ranges

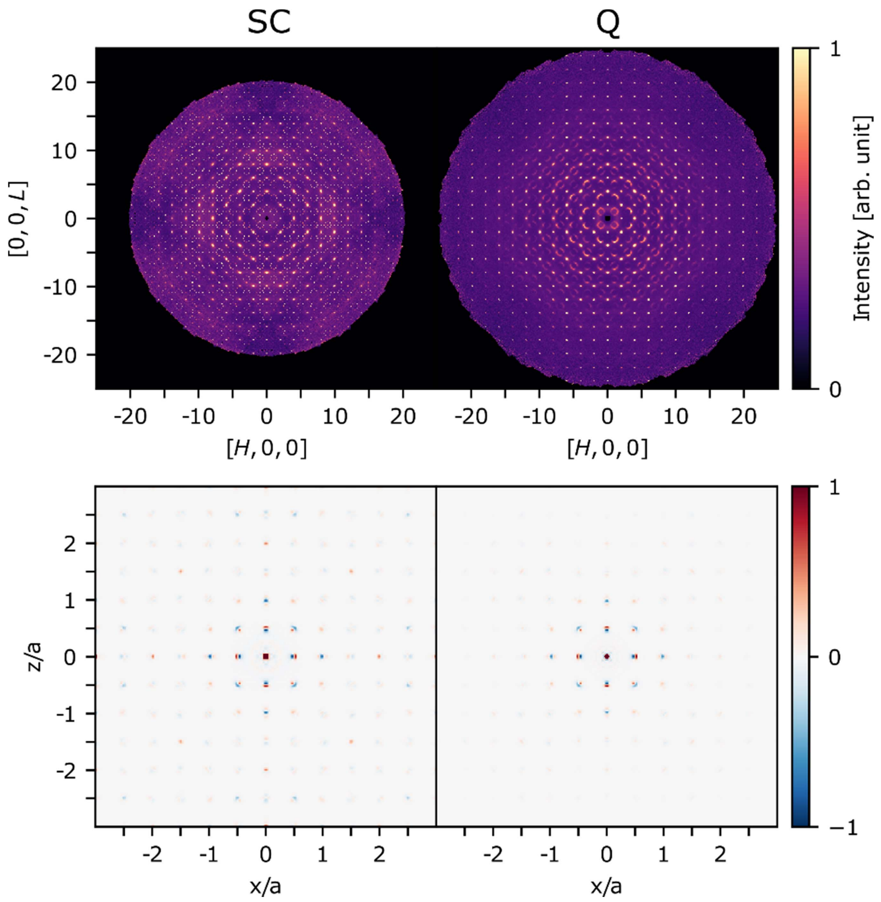

Figure S4 The measured scattering in the H0L plane and the 3D-ΔPDF in the 010 plane for the two representative samples shown with a larger range.

S5. Different ground state candidates

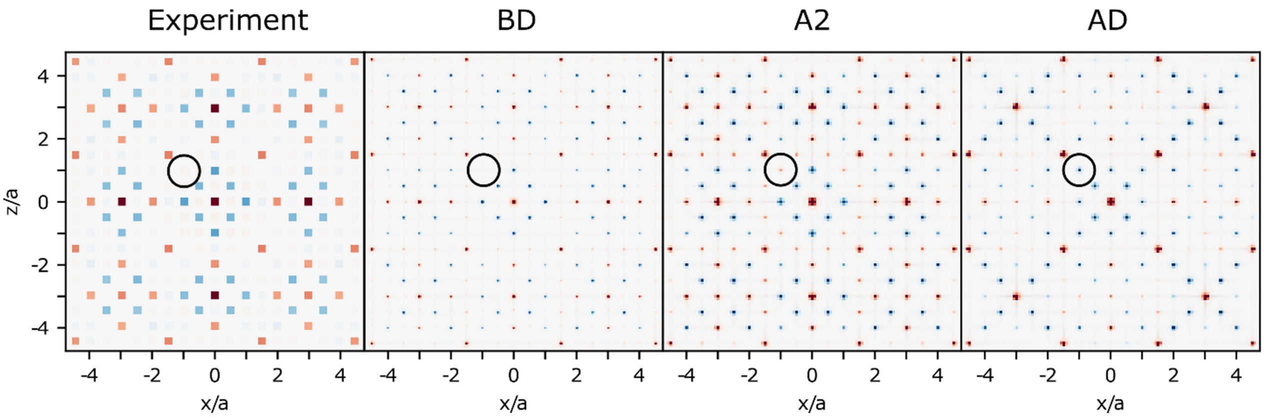

Figure S5 Integrated 3D-ΔPDF for experimental data compared with calculated 3D-□PDF for different ground state candidates.

In the vacancy repulsion model, vacancies on the Nb substructure avoid nearest and next-nearest pairs. For x = 1/6 there are several types of vacancy configurations that completely avoid nearest and next-nearest pairs (Roth *et al.*, 2020). Of these, three have scattering patterns consistent with the measurement, as was shown previously (Roth *et al.*, 2020). These are the A2, AD, and BD type ground states of the model. AD and BD are non-periodic and disordered.

The left panel of Figure S5 shows the integrated amplitudes from the measured data on the SC-0.81 sample, which had the sharpest additional peaks in the scattering pattern. The three panels to the right show the calculated 3D-ΔPDF maps in the 010 plane for the three candidate ground states of the vacancy repulsion model without Sb and Co relaxations.

Both the A2 and BD models are in general good agreement with the experiment, while the AD model has several discrepancies in the sign of amplitudes. The black circles marks a feature which is different for the three models. For the BD model it is zero, for the A2 model it is positive and for the AD model it is negative. In the experiment this integrated amplitude is approximately zero, but weakly positive, suggesting the "BD" model to be the most accurate. However, there are still some differences between the measurement and BD model, suggesting either the BD model is not the complete description, or that the peak integration method is not accurate enough.

S6. Calculated scattering

Figure S6 shows the scattering in the H0L and HHL planes for one of the induction furnace samples together with several ground state models with vacancy repulsion and relaxation of Co and Sb. The top row shows the measured scattering in the SC-0.81 sample. The second row shows the calculated scattering for the BD ground state. Many of the features observed in the measurement are also reproduced by the model. This includes the 8 point rings of sharp scattering and some of the intensity modulations. The intensity modulation of the rings marked by green, red and blue circles are reproduced quite well. However, there are also clear differences when comparing these. The measured data shows peaks inside the 8-point rings, which is not in the current model scattering. See for example the ring marked by the white circle. As was shown in the previous paper (Roth *et al.*, 2020), the peaks inside the ring can be explained by the vacancy repulsion model for $x > 1/6$ and $J_2/J_1 < 0.5$. The third row in Figure S6 shows the calculated scattering for a simulation with $x = \frac{1}{5}$, and $J_2/J_1 = 0.45$. This produces the additional peaks inside the rings. However, they are much weaker than in the experiment and not very visible in the figure. The fourth row shows the same simulated data with a 10% colour range compared to the third row to visualize the additional peaks better. The weakness of the peaks together with the following discussion suggest that the additional peaks in the experiment come from a different origin. The SC samples refine to stoichiometries of $Nb_{0.822}CoSb$ and $Nb_{0.820}CoSb$, suggesting the deviation from $x = 1/6$ to be quite small, if significant at all. With such small deviations the model does not produce clear peaks at the ring center. Furthermore, the peaks inside the 8-point rings in the measured data seem to become stronger with increasing q, while they decreases with q in the simulated data (see the blue, yellow and white circles). This could suggest that there is another reason for the peaks inside the rings. There are also further weak peaks in the measured scattering from the SC sample, not currently explained by the model, showing that there is still more to learn about these samples.

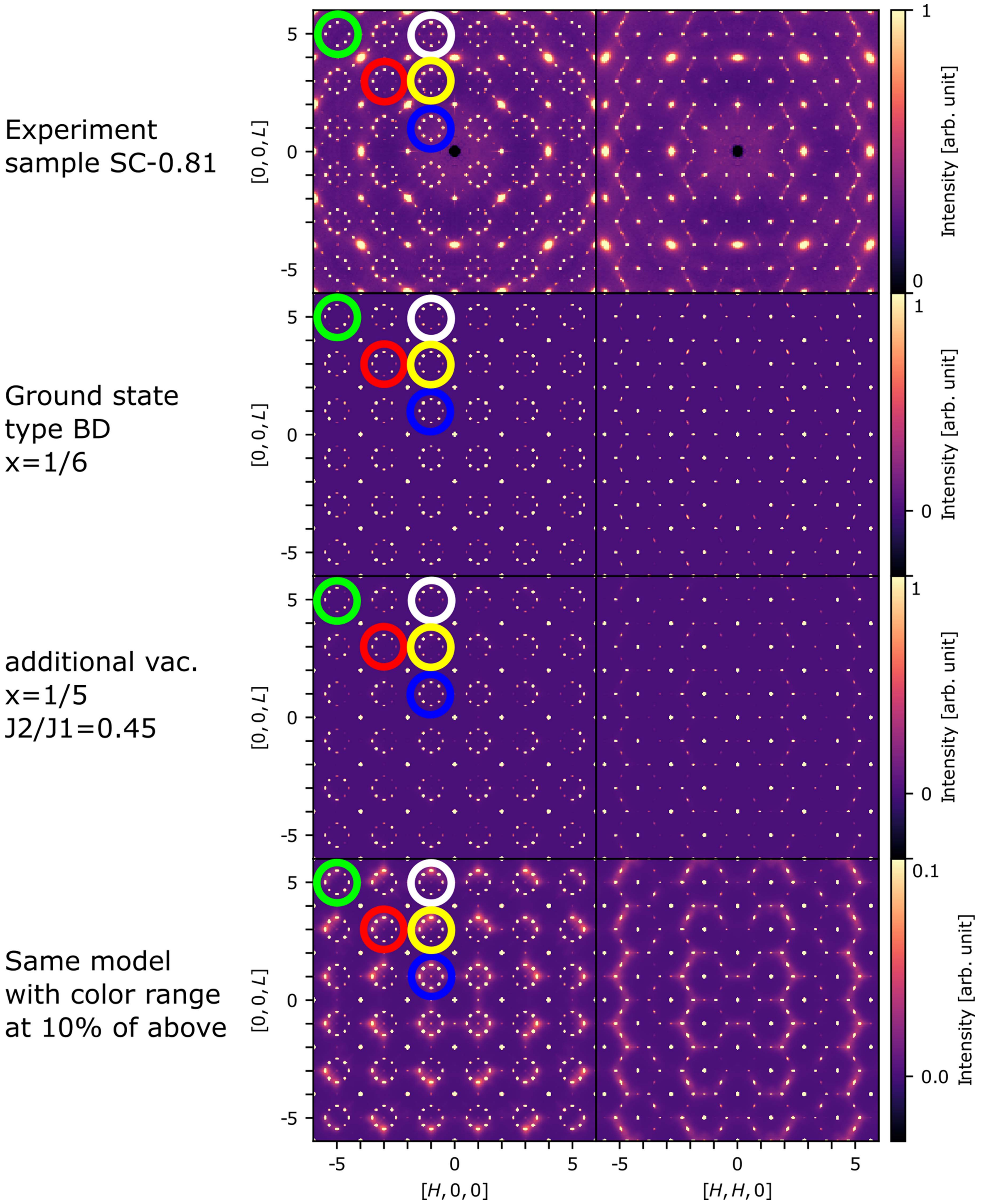

Figure S6 Experimental data and calculated scattering for several simulated ground-state models.

S7. Comments on the paper by Nan *et al.* (2020)

A study by Nan *et al*. looks at high-angle annular dark-field imaging (HAADF), a type of high-resolution scanning transmission electron microscopy (STEM) measured on a sample of nominal composition $Nb_{0.8}CoSb$ (Nan *et al.*, 2020). The HAADF is measured along the [110] zone axis where there is no blocking of elements of different kinds. Based on this it identifies that three different amplitudes correspond to the three different elements. It then tries to show that the short-range order is mainly displacive by making Fourier transforms of the measured and ideal amplitudes together with the measured and ideal positions of atoms.

The problem is that HAADF cannot see the vacancies. The transmission-type measurement gives a projection down along the 110 zone axis. This means that when a vacancy is present on a Nb position, the measurement will still see the Nb from other layers, as not all Nb down along a column will be missing. This is clear when looking at figure 2b in their paper. Clearly a dot is seen for every Nb position, as it is a projection along 110, giving an average over many layers. Because they cannot see the vacancies but only some degree of the displacements, they reach the conclusion: "*We found that the short-range order is predominantly displacive.*" However, this is meaningless as their measurement does not allow analysis of the compositional disorder.

If the short-range order in the ED pattern was predominantly displacive, it would also not be compatible with the measured ED pattern (Figure 1a in their paper). Displacive short-range order gives diffuse scattering which is very weak close to the beam center and increases away from the beam center, whereas compositional short-range order gives diffuse scattering which is more constant in intensity (for x-ray scattering it will decrease slowly away from the beamcenter because of the atomic scattering factor). This is illustrated in figure S7.

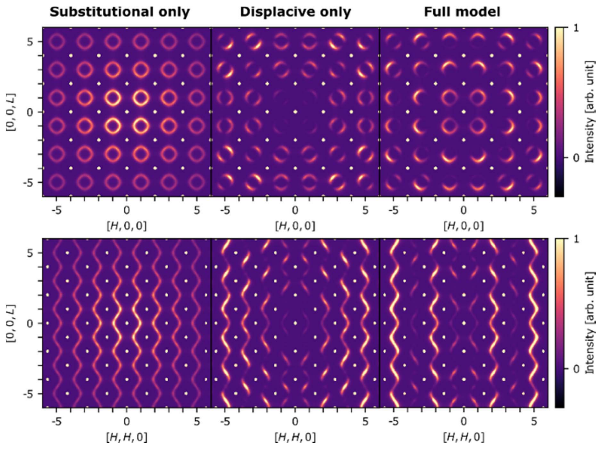

Figure S7 Calculated scattering for substitutional only (left), displacive only (center) and combined models (right).

The left column of Figure S7 shows the calculated diffuse scattering for the Nb/vacancy order, without any movements of Sb and Co. This gives diffuse scattering which decreases slowly in intensity due to the x-ray scattering factor. The central column shows diffuse scattering with only the Sb and Co displacements without any contribution from Nb. This gives diffuse scattering, which is very weak close to the center and increases away from the center. The right column shows the full model with both the Nb/vacancy order and Sb and Co displacements, giving the best agreement with experiment.

The paper (Nan *et al.*, 2020) avoids mentioning that the electron-diffraction data had already been explained to a higher accuracy using just substitutional short-range order (Roth *et al.*, 2020), even though the paper cites Roth *et al.* (2020) in another context, and re-uses several references that were first linked to the $Nb_{1-x}CoSb$ system in that paper.
